# Hot needles can confirm accurate lesion sampling intraoperatively using [^18^F]PSMA-1007 PET/CT-guided biopsy in patients with suspected prostate cancer

**DOI:** 10.1007/s00259-021-05599-3

**Published:** 2021-11-02

**Authors:** Daniela A. Ferraro, Riccardo Laudicella, Konstantinos Zeimpekis, Iliana Mebert, Julian Müller, Alexander Maurer, Hannes Grünig, Olivio Donati, Marcelo T. Sapienza, Jan H. Rueschoff, Niels Rupp, Daniel Eberli, Irene A. Burger

**Affiliations:** 1 Department of Nuclear Medicine, University Hospital Zurich, University of Zurich, Zurich, Switzerland; 2 Department of Radiology and Oncology, Faculdade de Medicina FMUSP, Universidade de Sao Paulo, Sao Paulo, Brazil; 3 Nuclear Medicine Unit, Department of Biomedical and Dental Sciences and Morpho-Functional Imaging, University of Messina, Messina, Italy; 4 Department of Urology, University Hospital Zürich, University of Zurich, Zurich, Switzerland; 5 Department of Diagnostic and Interventional Radiology, University Hospital Zurich, University of Zurich, Zurich, Switzerland; 6 Department of Pathology and Molecular Pathology, University Hospital Zurich, University of Zurich, Zurich, Switzerland; 7 Department of Nuclear Medicine, Kantonsspital Baden, Baden, Switzerland

**Keywords:** Biopsy guidance, Primary staging, PSMA PET, Targeted biopsy, Template biopsy

## Abstract

**Purpose:**

Prostate-specific membrane antigen (PSMA)-targeted PET is increasingly used for staging prostate cancer (PCa) with high accuracy to detect significant PCa (sigPCa). [^68^ Ga]PSMA-11 PET/MRI-guided biopsy showed promising results but also persisting limitation of sampling error, due to impaired image fusion. We aimed to assess the possibility of intraoperative quantification of [^18^F]PSMA-1007 PET/CT uptake in core biopsies as an instant confirmation for accurate lesion sampling.

**Methods:**

In this IRB-approved, prospective, proof-of-concept study, we included five consecutive patients with suspected PCa. All underwent [^18^F]PSMA-1007 PET/CT scans followed by immediate PET/CT-guided and saturation template biopsy (3.1 ± 0.3 h after PET). The activity in biopsy cores was measured as counts per minute (cpm) in a gamma spectrometer. Pearson’s test was used to correlate counts with histopathology (WHO/ISUP), tumor length, and membranous PSMA expression on immunohistochemistry (IHC).

**Results:**

In 43 of 113 needles, PCa was present. The mean cpm was overall significantly higher in needles with PCa (263 ± 396 cpm) compared to needles without PCa (73 ± 44 cpm, *p* < 0.001). In one patient with moderate PSMA uptake (SUV_max_ 8.7), 13 out of 24 needles had increased counts (100–200 cpm) but only signs of inflammation and PSMA expression in benign glands on IHC. Excluding this case, ROC analysis resulted in an AUC of 0.81, with an optimal cut-off to confirm PCa at 75 cpm (sens/spec of 65.1%/87%). In all 4 patients with PCa, the first or second PSMA PET-guided needle was positive for sigPCa with high counts (156–2079 cpm).

**Conclusions:**

[^18^F]PSMA-1007 uptake in PCa can be used to confirm accurate lesion sampling of the dominant tumor intraoperatively. This technique could improve confidence in imaging-based biopsy guidance and reduce the need for saturation biopsy.

**Trial registration number:**

NCT03187990, 15/06/2017.

**Supplementary Information:**

The online version contains supplementary material available at 10.1007/s00259-021-05599-3.

## Introduction

Prostate-specific membrane antigen (PSMA) positron emission tomography (PET) is a well-established technique for the assessment of prostate cancer (PCa) biochemical recurrence (BCR) [1] with a significant impact on patient management due to high sensitivity and specificity [2]. Further, [^68^ Ga]PSMA-11 PET showed substantial results in the intermediate- to high-risk PCa primary detection [3, 4] and initial staging before definitive therapy, even though current clinical guidelines do not yet recommend this application [5]. However, most of the studies are retrospective and composed of intermediate to high-risk PCa patients [3–6]. These tumors are more likely to overexpress PSMA; therefore, a potential bias and overestimated accuracy is possible. [^68^ Ga]PSMA-11 PET may also represent a useful tool to improve the accuracy of imaging-guided biopsy in PCa [7], even if data are still limited, and [^68^ Ga]PSMA-11 PET-biopsy guidance is nowadays recommended only in patients with previous negative biopsy [8]. In a comparative study, [^68^ Ga]PSMA-11 PET/computed tomography (CT)-guided biopsy detected more clinically “significant PCa” (sigPCa) than ultrasound (US)-guided biopsy [9], reaching a high per-patient accuracy of 81–93% for [^68^ Ga]- or [^18^F]PSMA PET-guided biopsy, with a slightly lower lesion-based accuracy ranging between 70 and 80% [10, 11]. There are different tracers available for PSMA PET imaging with slight differences in bio-distribution. The first tracer with broad application was [^68^ Ga]PSMA-11; with a high urinary excretion, this tracer sometimes limited the detection of local recurrence. The now also available tracer [^18^F]PSMA-1007 is excreted more over the hepatobiliary path and reduces spill over in the pelvis; on the other hand, a higher number of unclear bone uptake are still considered limiting the specificity for bone lesions [12]. For the detection of primary tumors, however, there are no published reports about differences between both tracers. When using [^68^ Ga]PSMA-11 PET/magnetic resonance imaging (MRI), a high correlation between sigPCa on saturation biopsy has been reported with a sensitivity of 96%; however, [^68^ Ga]PSMA-11 PET/MRI-targeted needles only reached a suboptimal sensitivity of 65% [13]. This is probably due to the difficulties in image-guided biopsy with the inherent risk to miss the target. An instant intraoperative confirmation therefore may further increase the precision of tissue sampling and physicians’ confidence. Due to the high accumulation of [^68^ Ga]/[^18^F]PSMA in sigPCa, we assumed that an intraoperative confirmation of high photon counts in the biopsy core could be used as a prompt confirmation for needle allocation within the targeted lesion.

## Materials and methods

### Study design

**Fig. 1:**
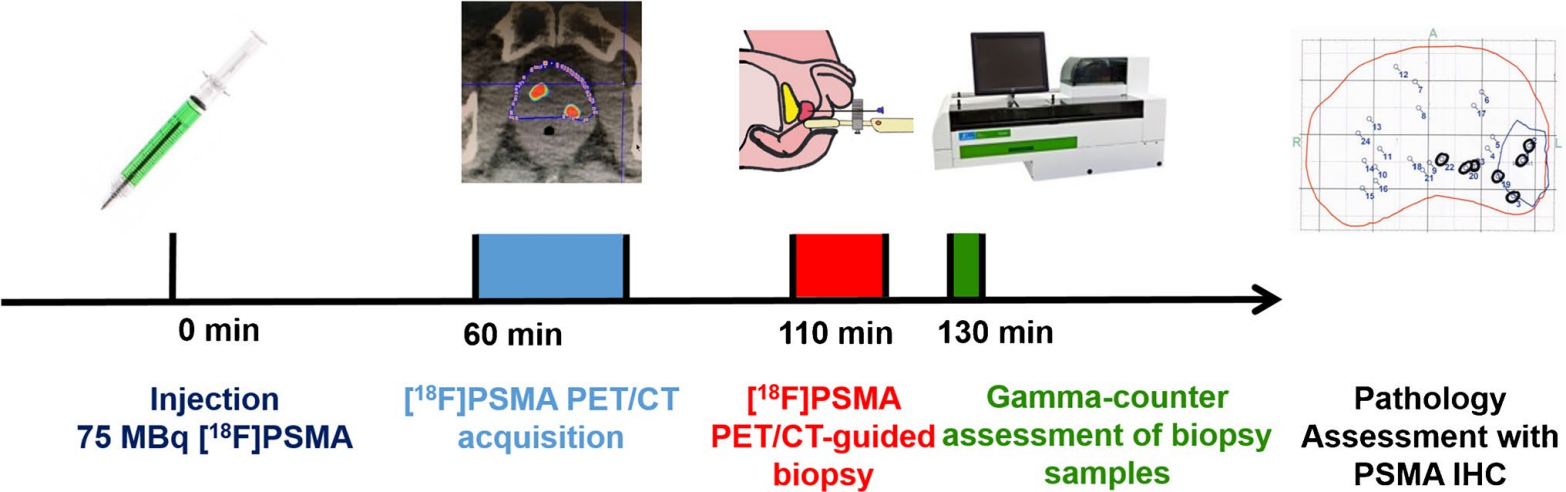
Illustration of the study workflow with time line for [^18^F]PSMA-1007 PET/CT-guided biopsy and uptake quantification

The study was designed as an open-label, single-center, non-randomized, proof-of-concept prospective investigation including patients with suspicious PCa. Patients with suspicion of cancer due to persistently elevated prostate-specific antigen (PSA) > 4 ng/mL, at least one suspicious lesion on multiparametric (mp) MRI (PiRADS 3 or more), and no prior biopsy were included. This study was approved by the institutional review board (BASEC Nr: 2017–00,016), was carried out following principles enunciated in the current version of the Declaration of Helsinki, and is registered in the international trial registry ClinicalTrials.gov (NCT03187990). To increase the potential time frame of photon count detection for this project, we amended the study protocol and used [^18^F]PSMA-1007 with a 109.8-min half-life, instead of [^68^ Ga]PSMA-11 with only 67.7 min. Furthermore, due to logistics and proximity between PET/CT scanner and biopsy suite, we changed the imaging modality from PET/MRI to PET/CT, using an ultralow dose CT for attenuation correction over the pelvic region. Exclusion criteria were in analogy to the previously published paper [13]: patients age < 30 and > 80; prostate biopsy within 8 weeks before the study; patients with previous pelvic irradiation, transurethral resection of the prostate (TURP), or androgen deprivation hormonal therapy (ADT); and patients with any contraindication for prostate biopsy as well as patients with active urinary tract infection or indwelling catheter. All patients signed a dedicated informed consent. We included 5 patients between 01.09.2020 and 30.11.2020, who underwent [^18^F]PSMA-1007 PET/CT followed by PET/CT-guided and section-based saturation template biopsy. After the biopsy, the samples were transferred immediately to a gamma spectrometer to measure the PSMA accumulation in counts per minute (cpm). Counts were then correlated with histopathology in terms of WHO/ISUP grade group (GG), tumor length, SUV_max_ from the targeted lesion, and membranous PSMA expression on immunohistochemistry (IHC). The pathological assessment was considered as the standard of reference for tumor presence. In Fig. 1, we graphically represented the study workflow.

### [^18^F]PSMA-1007 PET/CT imaging acquisition and analysis

All patients underwent a pelvic PET/CT (two-bed positions) on a dedicated hybrid scanner (Discovery MI PET/CT, GE Healthcare, Waukesha, WI, USA) 60 min after injection of 78 ± 0.9 MBq (77–79) of [^18^F]PSMA-1007. Details of the imaging protocol are given in the Supplementary materials. For the biopsy-targeting purpose, suspected lesions were delineated on [^18^F]PSMA-1007 PET/CT by a double-boarded physician (nuclear medicine and radiologist) with 2–3 years of experience in PSMA PET imaging (AM, HG). [^18^F]PSMA-1007 PET/CT images were fused on PMOD (PMOD v3.8, PMOD-Technologies LLC), through which all CT and reconstructed PET images could be viewed side by side by the reader as well as in fused mode. We used the color scale “rainbow” with a window adapted to allow clear visualization of the target lesion in contrast to the background, thus increasing the conspicuity of suspicious lesions.

### Biopsy

Biopsies were performed under general anesthesia, by a specialized urologist (DE) with the US-MRI software fusion BiopSee® system. The software can work with any DICOM image, and therefore, fused axial [^18^F]PSMA-1007 PET/CT images in DICOM format were uploaded to the BiopSee system instead of the T2-weighted MRI sequences. Standard trans-perineal template biopsy with the number of cores adapted to the prostate volume, as well as PSMA-fused-PET/CT-targeted biopsy, was performed with a maximum of three cores per target lesion. Patients with no suspicious uptake on PSMA PET/CT or with discordant lesions between PSMA PET/CT and mpMRI underwent template biopsy, and the urologist was free to additionally target any suspicious lesion on mpMRI. Exposure rates were measured at 10 cm and 1 m distance from the patient 3 h after injection before the first biopsy, to assure radiation exposure safety for the team at the operating room.

### Biopsy core activity quantification

For the measurement and quantification of the biopsy cores, a WIZARD^2^ 2480 automatic gamma counter (PerkinElmer Inc., Waltham, MA, USA) was used [14]. The counter was calibrated with 1 ml of 500 Bq/ml ^18^F inside a glass vial as a starting concentration. The measurement time was 1 min, and the measurements were repeated every 10 min until the expected concentration reached 100 Bq/ml. Background correction was applied manually by measuring 3 glass vials filled with 1 ml of water and taking the average. The measurements were given in cpm, and the energy window was set to 511 keV ± 10% (460–562 keV). By linear regression of the measured against the expected activities, a calibration factor of 2.767 (cpm/Bq) was calculated (36.14% sensitivity). Each biopsy sample acquired by the surgeon was placed in a glass vial in a solution of 10 ml of formalin. The spectrometer counting started 10 min after the biopsy. Vendor-provided racks could hold up to 5 hemolysis tubes. The raw spectra were extracted by vendor-specific software and analyzed on Microsoft Excel (Office 2019, Microsoft Corporation). Biopsy cores were then directly transferred for histopathology assessment.

### Reference standard

The pathological assessment was considered as the standard of reference for tumor presence. SigPCa was defined as ISUP GG ≥ 3. Results of [^18^F]PSMA-1007 PET targeted biopsies were compared to the template biopsies regarding the presence of PCa on pathology; for discrepant cases (e.g., high cpm without evidence of tumor or low counts despite significant cancer), PSMA expression was visually assessed with PSMA IHC for intensity, using a four-tiered score (0 = negative, 1 +  = weak, 2 +  = moderate, 3 +  = strong) and percentage of tumor area without PSMA expression. The analyses were performed by two board-certified genitourinary pathologists (NR, JHR) with 10 and 12 years of experience, following the WHO2016/ISUP prognostic GG guidelines [15]. Technical PSMA staining details have been published previously [16].

### Data analysis

Statistical analyses were performed using SPSS statistics software, version 26 (IBM). Descriptive analyses were used to display patient data as mean and range; frequency distribution with percentages was used to summarize categorical variables, and means with standard deviations or medians with interquartile ranges (IQR) were used to describe continuous variables. The Mann–Whitney test was used to compare mean values. Spearman rank test was used to correlate counts with histopathology in terms of WHO/ISUP grade. The optimal cut-off to predict the presence of tumor based on cpm was assessed with receiver operating characteristics (ROC) analysis and calculation of the area under the ROC curve (AUC). The optimal cut-off for the ROC curve was determined by applying the Youden index (maximum of sensitivity + specificity − 1). Statistical analyses were performed by IAB, DAF, and RL (nuclear medicine physicians).

## Results

**Fig. 2:**
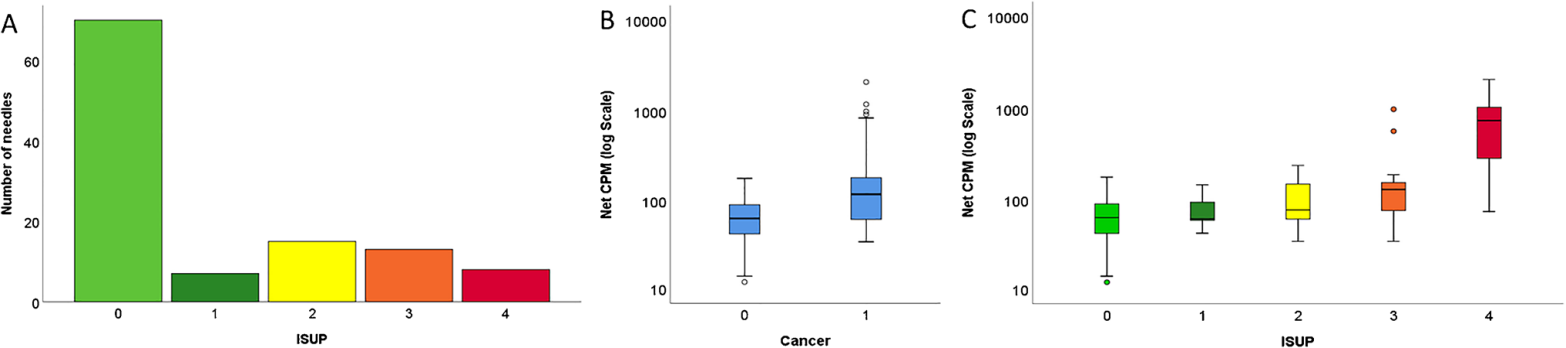
**A** Bar graph for the number of needles for each biopsy ISUP grade. **B** Box plot illustrations for cpm distribution (logarithmic scaling) according to the absence (0) or presence (1) of PCa. **C** Box plot illustrations for cpm distribution in needles according to biopsy ISUP grade

In this proof-of-concept study, five consecutive patients (median age 62 years old, IQR 59–71.6; median PSA 7 ng/ml, IQR 6.1–26.5) underwent [^18^F]PSMA-1007 PET/CT-targeted biopsy with a mean of 51.6 days (14–169) after mpMRI. The mean time interval between PSMA PET/CT and biopsy was 3.1 ± 0.3 h (range 2.5–3.5). Patient characteristics are given in Table 1. In 4 of 5 patients (80%), sigPCa was present with a maximum ISUP of 3 or 4. A total of 118 biopsy cores were sampled; due to technical problems, 5 biopsy cores from patient 1, negative for PCa, were not analyzed in the gamma counter and therefore not considered for the whole analysis. Forty-three of 113 cores (38%) resulted positive for PCa at pathology, defined as any ISUP GG ≥ 1. Needles distribution according to ISUP GG was as follows, 7/43 ISUP1, 15/43 ISUP2, 13/43 ISUP3, and 8/43 ISUP4, as shown in Fig. 2A.

**Table 1:** Patients characteristics

Pat	Age	PSA (ng/ml)	PiRADS	SUV_max_ (SUV_mean_)	mpMRI (ADC_mean_)	ISUP_max_ on biopsy	Max PCa length (mm)*	Positive/total cores (*n*)
1	79	6.3	5	17.0 (10.3)	0.893	3	6.5	4/24
2	69	7	5	8.3 (4.7)	0.585	3	10.6	9/23
3	62	13.2	4	17.1 (9.7)	0.587	3	5	14/24
4	61	5.7	5	8.7 (4.7)	MM	0	0	0/24
5	53	66.5	5	104.5 (59.5)	0.678	4	11	13/23

**Legend:** *PSA*, prostate-specific antigen; *PiRADS*, prostate imaging reporting and data system; *SUV*, standardized uptake value; *mpMRI*, multiparametric magnetic resonance imaging; *ADC*, apparent diffusion coefficient; *MM*, mismatch between mpMRI and PSMA PET; *ISUP*, International Society of Urological Pathology/WHO2016 Gleason score prognostic grade group; *PCa*, prostate cancer; *on a single biopsy core

In one patient (patient 4), no PCa was detected despite diffuse moderate tracer uptake on [^18^F]PSMA-1007 PET (SUV_max_ 8.7) and suspicious lesions on mpMRI (PiRADS 5); in this patient, 13 out of 24 needles had intermediate to high cpm ranging between 100 and 200 but only signs of inflammation at pathology, with 11/13 needles with confirmed PSMA expression on IHC. In all 4 patients with PCa, the lesions detected on mpMRI and PSMA PET/CT were concordant. On biopsy, the first (3 of 4) or second (1 of 4) PSMA PET-guided needle detected sigPCa, with high cpm ranging from 156 to 2079 cpm, as shown in Table 2.

**Table 2:** **“**Hot-needle” characteristics

Patient	Needle	cpm	Gleason on first sig. PCa core (ISUP)	PCa length on first sig. PCa core (mm)	Maximum Gleason on template (ISUP)
1	First	568	4 + 3 (3)	6.5	4 + 3 (3)
2	First	156	4 + 3 (3)	3.2	4 + 3 (3)
3	Second	224	3 + 4 (2)	5	4 + 3 (3)
5	First	2079	4 + 4 (4)	9	4 + 4 (4)

**Legend**: *cpm*, counts per minute; *ISUP*, International Society of Urological Pathology/WHO2016 Gleason score prognostic grade group; *PCa*, prostate cancer

Exposure rates at 10 cm and 1 m distance from the patient were < 2 µSv/h and < 0.1 µSv/h, respectively, 3 h after injection of a median dose of 78 MBq of [^18^F]PSMA-1007.

### Positive needles versus negative needles

**Fig. 3:**
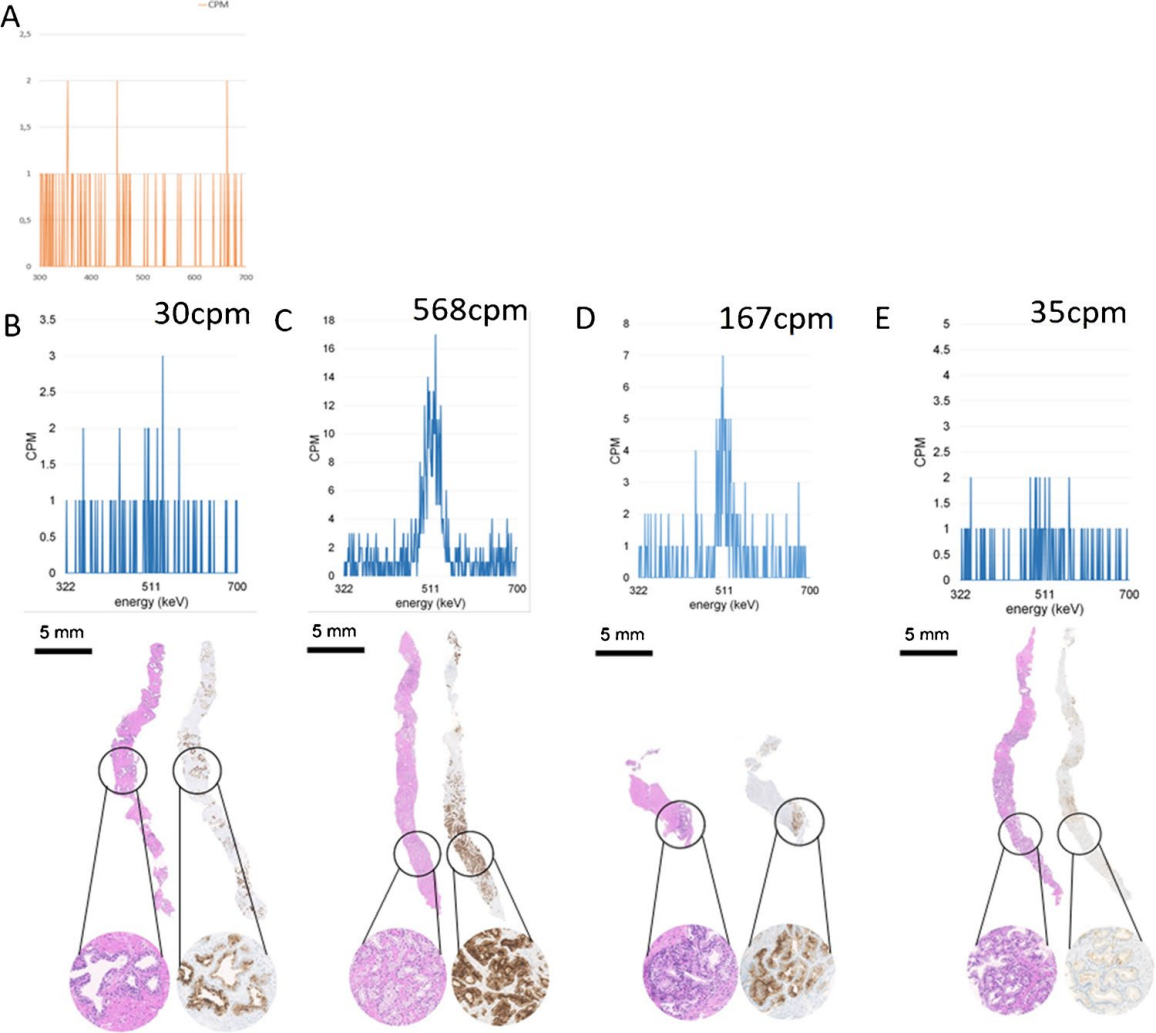
**A** Gamma spectrometer assessment of the background. **B** True negative: patient 1, needle 11; low uptake on [^18^F]PSMA-1007 PET/CT (SUV_max_ 5.8) and low counts per minute (cpm = 30) were also negative for PCa on histopathology. **C** True positive: patient 1, needle 1; intense uptake on PSMA PET/CT (SUV_max_ 17.0) leads to a target biopsy with high counts per minute (cpm = 568), with confirmed PSMA-positive ISUP GG 3 PCa on histopathology.** D** False positive: patient 4, needle 8; focal uptake on PSMA PET/CT (SUV_max_ 8.7), corresponding to high counts per minute (cpm = 167) without PCa but inflammation and PSMA-positive benign glands on histopathology. **E** False negative: patient 2, needle 22; no increased PSMA uptake on PET/CT (SUV_max_ 5.65), corresponding to low counts per minute (cpm = 35) but confirmed ISUP GG 3 PCa on histopathology without PSMA expression on IHC

The median and mean cpm were overall significantly higher in needles with PCa (119 cpm; mean 263 cpm ± 395.7) compared to needles without PCa (64 cpm; mean 73 ± 44, *p* < 0.001), as shown in Fig. 2B. Further, we observed a positive correlation between ISUP grade at pathology and cpm (*r* = 0.431, *p* < 0.001), as shown in Fig. 2C. In Fig. 3, we report examples for true and false positive/negative needles, with the results from the gamma counter, the corresponding histopathology, and PSMA IHC.

### Intraoperative prediction of PCa biopsy based on cpm

Without considering patient 4, we assessed the potential of biopsy core cpm to intraoperatively detect PCa. The resulting AUC for cpm was 0.81 for PCa detection, with an optimal cut-off at 75 cpm, yielding a sensitivity and specificity of 65.1% and 87%, respectively (Fig. S1).

### False negative and false positive cases

On per-core analysis, 61/113 core biopsies counted less than 75 cpm. Among them, 15/61 were false negative with PCa detected on histopathology (25%); these lesions had a mean cancer length of 2.0 ± 2.0 mm, and 10/15 had an ISUP > 1 (67%), as shown in Table S1. On the other hand, 52 of 113 core biopsies counted more than 75 cpm. Among them, 22 (42%) were false positive without PCa at histopathology. However, 16 of these 22 false positive cores came from patient 4 with acute inflammation, as shown in Table S2.

**Fig. 4:**
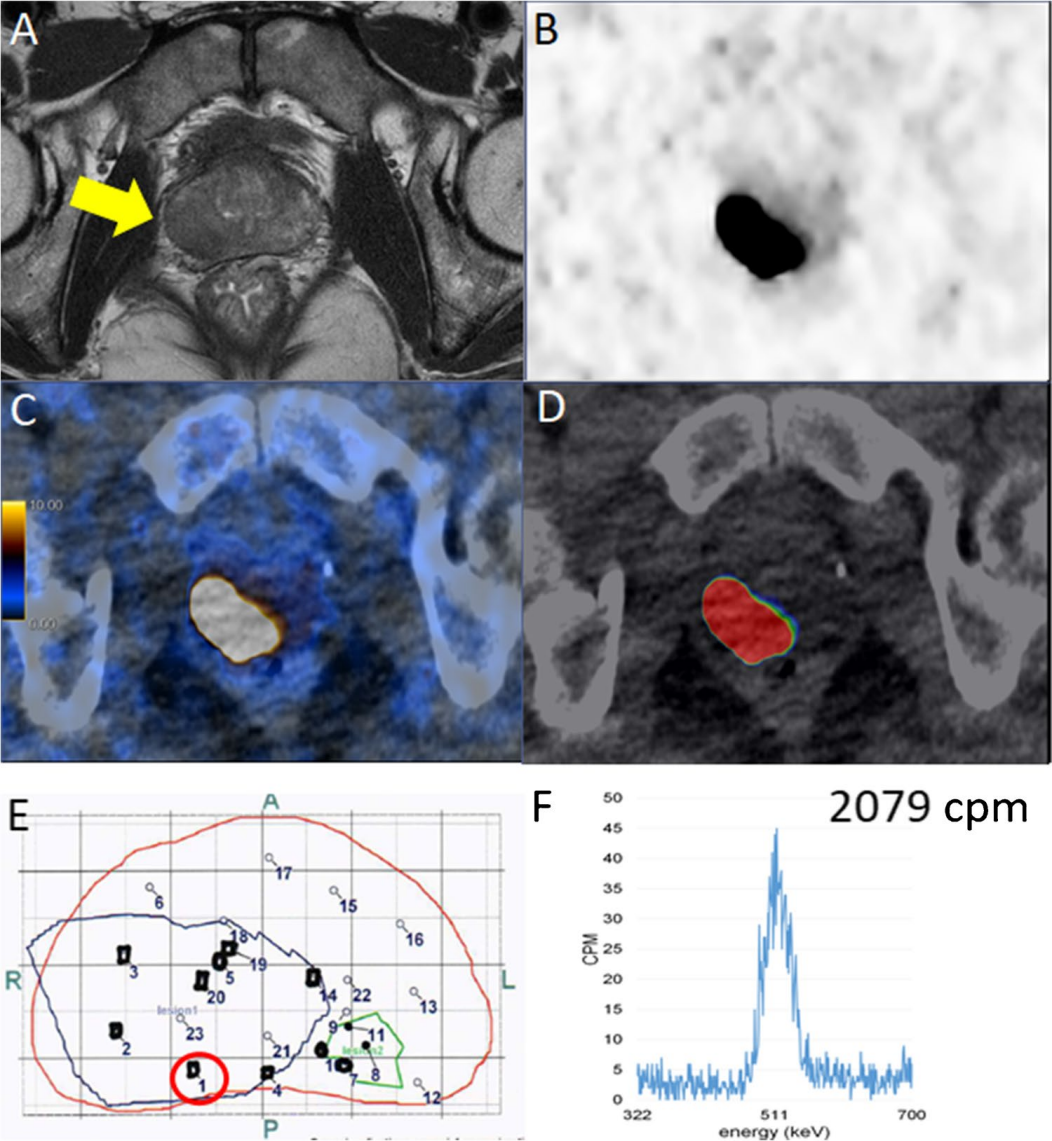
Fifty-year-old patient 5 with a PSA of 66.5 ng/ml. **A** mpMRI detected a suspicious lesion with hypointense signal on T2-weighted imaging on the right peripheral zone (yellow arrow). **B**–**C** PET/CT confirmed high [^18^F]PSMA-1007 tracer uptake (SUV_max_ 104.5) in the same lesion. **D** The fused PSMA PET/CT DICOM file prepared by the nuclear medicine physician who outlined the suspicious lesion on PET for biopsy targeting that was loaded to the BiopSee system. **E** Corresponding histopathology prostate map with biopsy targeted lesions delineated in blue and green according to the DICOM file. Each number in **E** represents a biopsy sample; the position of needles with clinically significant cancer is marked by black dots. The red circle highlights the first needle detecting sigPCa, with the corresponding high cpm (2079) from the spectrometer (**F**)

**Fig. 5:**
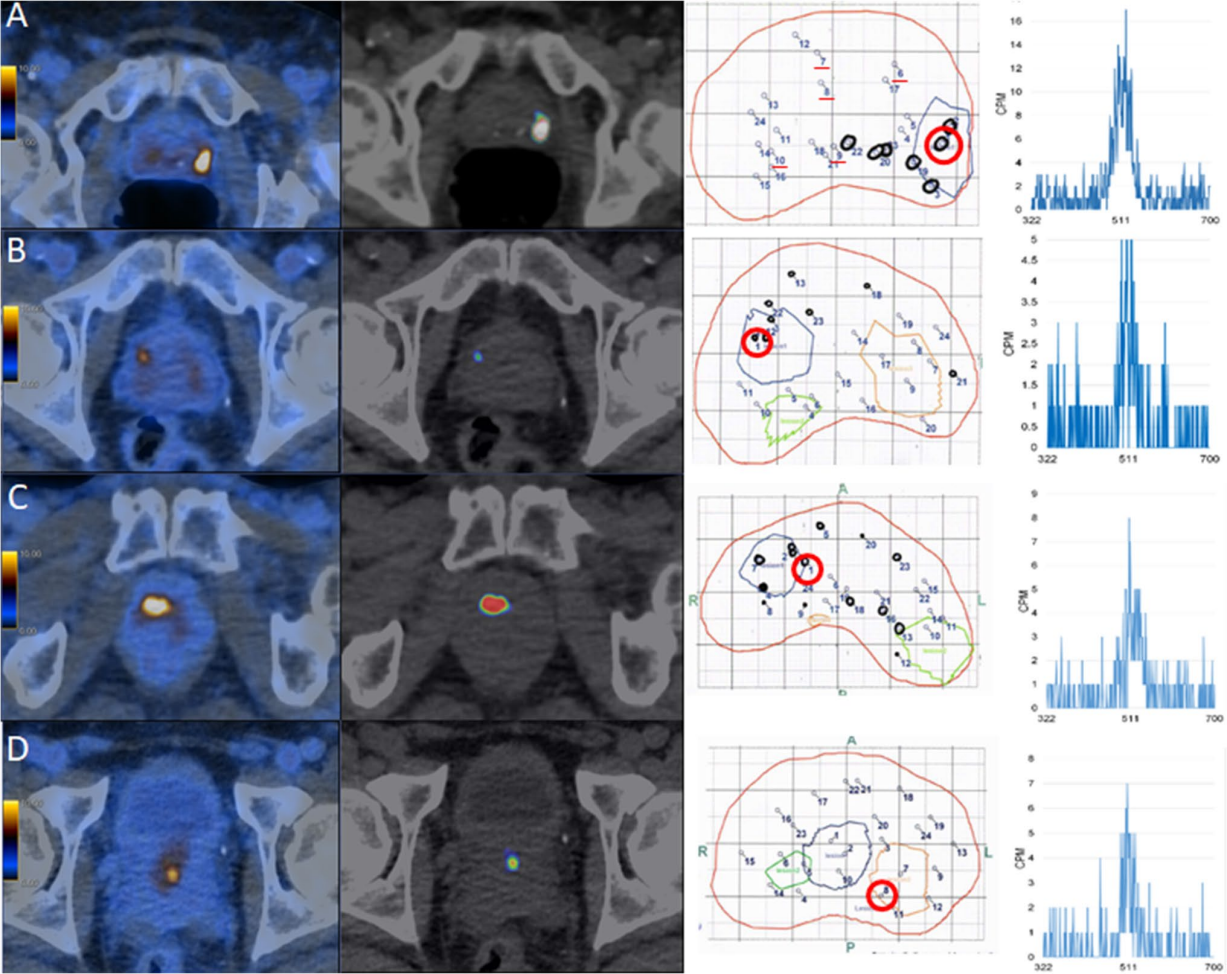
Summary images of patients 1–4. In the first column, the regular fused [^18^F]PSMA-1007 PET/CT images are given, using a PET window of 0–10. The second column represents the selected PET/CT DICOM set to be loaded to the BiopSee system. In the third column, the histopathology prostate map with targeted lesions is given. Biopsy needles with clinically significant cancer are marked by black dots. In the last column, the corresponding spectrometer results from the red circled biopsy in column three are given. **A** Patient 1 with tracer high tracer uptake on PET/CT (SUV_max_ 17.0). Underlined in red are the 5 not measured biopsy cores; the red circle highlights the first needle detecting sigPCa, with the corresponding high cpm (568) in the spectrometer. **B** Patient 2. PET/CT with moderate tracer uptake (SUV_max_ 8.26). The red circle highlights the first needle detecting sigPCa, with the corresponding high cpm (156) from the spectrometer. **C** Patient 3. PET/CT confirmed high tracer uptake (SUV_max_ 17.15). The red circle highlights the first needle detecting sigPCa, with the corresponding high cpm (224) from the spectrometer. **D** Patient 4 with inflammatory changes only: PET/CT showed moderate tracer uptake (SUV_max_ 8.71) in the transition zone, anterior to the mpMRI suspicious lesion. The red circle highlights the 8th needle with corresponding spectrometer results, showing some increased cpm (167) but no PCa

In Fig. 4, we illustrated the MRI and PET/CT findings with the corresponding pathology map, highlighting the first positive needle for patient 5, with the corresponding result from the gamma counter. Images for patients 1–4 are given in Fig. 5.

## Discussion

[^18^F]PSMA-1007 accumulation in biopsy cores of PCa lesions can be detected using a regular gamma counter for PET applications. False negative cores were not due to inadequate sensitivity of the gamma counter for PSMA accumulation but represented either ISUP GG 1 lesions, short-length, and PSMA negative tumor areas of more than 40% or a combination of them. Furthermore, with significantly higher cpm for higher ISUP grades, our results confirmed that PSMA expression and PSMA tracer accumulation correlated with tumor aggressiveness and that PCa shows higher SUV values than normal prostatic tissue [17, 18]. In one patient (patient 4) (Fig. 5), only inflammatory findings were detected on saturation biopsy despite suspicious results on mpMRI (PiRADS 5) and PSMA PET/CT (SUV_max_ 8.7). In this patient, biopsy cores had also moderate to high count rates in 16 of 22 needles, with mild to strong PSMA staining in benign glands on IHC (Fig. 3D), representing a case of inflammation as a potential reason for false positive PSMA PET findings, as has been described before [19].

On the other hand, 15 of 61 cores with low activity (< 70 cpm) were positive for PCa on saturation biopsy. Five of 15 cores were not clinical sigPCa with ISUP GG 1, 11 of 15 were smaller than 2 mm, and 4 cores were ≥ 60% PSMA negative on PSMA IHC. Despite the paucity of data in the literature regarding measurement of PSMA-labeled tracer activity in patients’ in vivo samples, interesting results have been described for Cerenkov luminescence imaging (CLI). A prospective study using CLI of [^68^ Ga]PSMA-11 for intraoperative detection of residual cancer or lymph node (LN) metastasis recently presented their safety data. Using an average of 122 MBq of [^68^ Ga]PSMA-11, the estimated dose equivalent for the surgeon was 0.5 µSv/h (0–1 µSv/h) for interventions starting 190 min after injection, concluding that the operational dose was neglectable [20]. This is in concordance to our results of a dose rate < 2 µSv/h and < 0.1 µSv/h in 10 cm and 1 m distance from the patient, respectively, 3 h after injection of a median dose of 78 MBq of [^18^F]PSMA-1007, despite using an isotope with longer half-life. Interesting results for CLI have been also described for [^68^ Ga]PSMA administration before surgery in the detection of positive surgical margins, even if CLI in the base of the prostate can generate false positive signals [21, 22]. Also, the use of [^111^In]PSMA and [^99m^Tc]PSMA have been described for surgical guidance, facilitating the detection of small LN and revealing additional lesions [23–26]. Such radioguided procedures were based on the detection of γ-photons with handheld probes; however, due to the high penetration depth of background signals from tracers accumulating in other organs, accurate detection can be hampered. Furthermore, for biopsy guidance purpose, the lower image resolution of scintigraphy in comparison to PET images makes single-photon emission technique less attractive. Nonetheless, no data regarding intraoperative counting of biopsy cores after PSMA PET/CT-guided biopsy have been described so far. Therefore, this is the first study showing that the activity in biopsy cores after PSMA PET/CT-guided biopsies is high enough to be detected and quantified with a dedicated gamma spectrometer. This is a simple and feasible technique that may increase the operator confidence for target selection with minimal additional radiation burden. Therefore, this approach could decrease the number of needed/sampled cores, ultimately leading to a reduction in side effects plus improved specificity.

The main limitation of this study is the small sample size due to the complex interaction between nuclear medicine, urology, and pathology that was in part due to the fact that no spectrometer was available in the operation room. With the COVID-19 pandemic, this exchange became even more restricted. Furthermore, this is a proof of principle that the activity of significant cancer in a biopsy is high enough to be detected with a gamma spectrometer; it is not answering the important question how and especially for which patients this technology should be used. However, given the excellent correlation between count rates of biopsy cores and tumor assessment on IHC, we believe that future investigations and investments for dedicated gamma counters are warranted.

In conclusion, [^18^F]PSMA-1007 uptake in PCa can be used to confirm accurate lesion sampling of the dominant tumor intraoperatively. This technique could improve confidence in imaging-based biopsy guidance and reduce the need for saturation biopsy.

## Supplementary Information

Below is the link to the electronic supplementary material.

Supplementary file1 (DOCX 32 KB)

## Data Availability

Data are available for bona fide researchers who request it from the authors.

## Abbreviations

ADT: Androgen deprivation hormonal therapy
AUC: Area under the ROC curve
BCR: Biochemical recurrence
CLI: Cerenkov luminescence imaging
Cpm: Counts per minute
CT: Computed tomography
GG: Grade group
IHC: Immunohistochemistry
LN: Lymph node
mp: Multiparametric
PCa: Prostate cancer
PET: Positron emission tomography
PSA: Prostate-specific antigen
PSMA: Prostate-specific membrane antigen
ROC: Receiver operating characteristics
SigPCa: Significant prostate cancer
TURP: Transurethral resection of the prostate
US: Ultrasound
MRI: Magnetic resonance imaging
