# Supplementary material for: Hot needles can confirm accurate lesion sampling intraoperatively using [18F]PSMA-1007 PET/CT-guided biopsy in patients with suspected prostate cancer
Source: Eur J Nucl Med Mol Imaging. 2021 Nov 2;49(5):1721–30. doi: 10.1007/s00259-021-05599-3 (PMC8560591; doi:10.1007/s00259-021-05599-3)
Supplement: Supplementary file 1 — Supplementary file1 (DOCX 32 KB) [file 259_2021_5599_MOESM1_ESM.docx]

**Supplemental material:**

**PET/CT imaging acquisition protocol**

PET data were acquired with time-of-flight (TOF) mode, followed by CT for attenuation correction, covering the same anatomical region. The PET acquisition consisted of 2-bed frames, each one 2 minutes long, with the following reconstruction parameters: OSEM - 3 iterations, 16 subsets, FWHMI of 6.3 mm, 1:4 Z-axis filter, and 6.4 mm Gaussian filter with both time-of-flight (TOF) and point spread function (PSF) modelling (OSEM_PSF_; VUE Point FX with SharpIR, GE Healthcare). For the CT attenuation correction, the tube voltage was set to 100 kV and automated dose modulation (range 60-440 mA/slice) was used. The pitch ratio was set to 1:0.984, and the slice thickness to 3.75 mm.

**Tables:**

**Table S1.** False negative cores characteristics

| **Grade (n)** | **Length (mm)** | **PSMA membrane staining intensity** | **PSMA negative area** | **cpm** |
| --- | --- | --- | --- | --- |
| **ISUP 3 (3)** | 2.6 | 2+ | 5% | 60 |
|  | 7.5 | 2+ | 60% | 35 |
|  | 2.1 | 1+ | 95% | 38 |
| **ISUP 2 (7)** | 1 | na | na | 45 |
|  | 1 | 1+ | 90% | 63 |
|  | 1 | 2+ | 10% | 63 |
|  | 6.3 | 1+ | 70% | 35 |
|  | 1.5 | 1+ | 40% | 69 |
|  | 1 | na | na | 61 |
|  | 1.3 | 1+ | 5% | 46 |
| **ISUP 1 (5)** | 1.5 | na | na | 66 |
|  | 1 | na | na | 59 |
|  | 1 | na | na | 43 |
|  | 1 | na | na | 61 |
|  | 1 | na | na | 62 |

**Legend**: PSMA prostate-specific membrane antigen; cpm counts per minute; ISUP International Society of Urological Pathology/WHO2016 Gleason score prognostic grade group; na not available.

**Table S2.** False positive cores characteristics

| **Patient (n)** | **PSMA membrane staining intensity** | **cpm** |
| --- | --- | --- |
| **1 (2)** | 2+ | 97 |
|  | 2+ | 91 |
| **2 (1)** | 3+ | 146 |
| **3 (1)** | 2+ | 88 |
| **4 (16)** | 1+ | 99 |
|  | nm | 121 |
|  | nm | 135 |
|  | 1+ | 132 |
|  | 3+ | 167 |
|  | 2+ | 91 |
|  | 2+ | 114 |
|  | 2+ | 129 |
|  | 1+ | 143 |
|  | 1+ | 161 |
|  | 1+ | 179 |
|  | 1+ | 148 |
|  | 1+ | 105 |
|  | 1+ | 147 |
|  | 1+ | 78 |
|  | 1+ | 175 |
| **5 (2)** | 3+ | 173 |
|  | 3+ | 80 |

**Legend**: PSMA prostate-specific membrane antigen; cpm counts per minute; nm not measurable


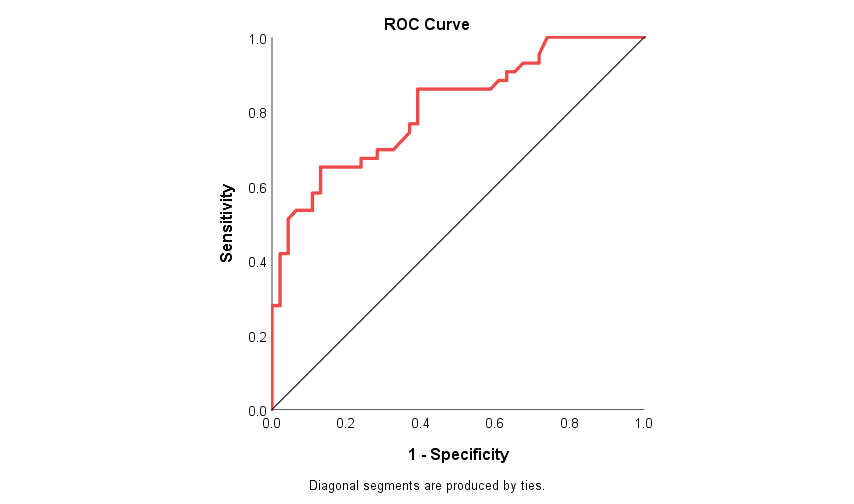


**Fig. S1** ROC curve analysis for cpm prediction of PCa. A cpm value of 75 reached sensitivity of 65.1% and specificity of 87%.
